# Osteoimmune Properties of Mesoporous Bioactive Nanospheres: A Study on T Helper Lymphocytes

**DOI:** 10.3390/nano13152183

**Published:** 2023-07-26

**Authors:** Laura Casarrubios, Mónica Cicuéndez, María Vallet-Regí, María Teresa Portolés, Daniel Arcos, María José Feito

**Affiliations:** 1 Departamento de Bioquímica y Biología Molecular, Facultad de Ciencias Químicas, Universidad Complutense de Madrid, 28040 Madrid, Spain; laura.casarrubios.molina@gmail.com (L.C.); portoles@quim.ucm.es (M.T.P.); 2 Instituto de Investigación Sanitaria del Hospital Clínico San Carlos (IdISSC), 28040 Madrid, Spain; mcicuendez@ucm.es; 3 Departamento de Química en Ciencias Farmacéuticas, Facultad de Farmacia, Universidad Complutense de Madrid, 28040 Madrid, Spain; vallet@ucm.es; 4 Instituto de Investigación Sanitaria Hospital 12 de Octubre i+12, 28040 Madrid, Spain; 5 CIBER de Bioingeniería, Biomateriales y Nanomedicina, CIBER-BBN, ISCIII, 28040 Madrid, Spain

**Keywords:** mesoporous bioactive glass, nanospheres, osteoimmunology, lymphocytes, ipriflavone, osteoporosis

## Abstract

Bioactive mesoporous glass nanospheres (nanoMBGs) charged with antiosteoporotic drugs have great potential for the treatment of osteoporosis and fracture prevention. In this scenario, cells of the immune system are essential both in the development of disease and in their potential to stimulate therapeutic effects. In the present work, we hypothesize that nanoMBGs loaded with ipriflavone can exert a positive osteoimmune effect. With this objective, we assessed the effects of non-loaded and ipriflavone-loaded nanoparticles (nanoMBGs and nanoMBG-IPs, respectively) on CD4^+^ Th2 lymphocytes because this kind of cell is implicated in the inhibition of osseous loss by reducing the RANKL/OPG relationship through the secretion of cytokines. The results indicate that nanoMBGs enter efficiently in CD4^+^ Th2 lymphocytes, mainly through phagocytosis and clathrin-dependent mechanisms, without affecting the function of these T cells or inducing inflammatory mediators or oxidative stress, thus maintaining the reparative Th2 phenotype. Furthermore, the incorporation of the anti-osteoporotic drug ipriflavone reduces the potential unwanted inflammatory response by decreasing the presence of ROS and stimulating intracellular anti-inflammatory cytokine release like IL-4. These results evidenced that nanoMBG loaded with ipriflavone exerts a positive osteoimmune effect.

## 1. Introduction

Research on the potential of nanomaterials for the treatment of osteoporosis has gained significant prominence in the last few years [1,2]. Nanoparticles can be designed to transport drugs to those regions of bone tissue for which drugs have low bioavailability. The development of porous nanoparticles capable of reversing osteoporosis by releasing siRNA was recently published [3]. On the other hand, the design of bioactive mesoporous nanoparticles (nanoMBGs) has demonstrated their ability to regenerate bone tissue as well as to release anti-osteoporotic agents at the intracellular level [4].

The effectiveness of nanoMBGs for the treatment of degenerative bone pathologies is attributed to two fundamental aspects. The first is their bone tissue regenerative activity, which is inherent to their chemical composition based on the SiO_2_-CaO-P_2_O_5_ or SiO_2_-CaO systems and to their mesoporous structure [5]. The second aspect is their ability to release drugs or therapeutic ions that can stimulate osteoblastic function and angiogenesis [6,7]. This is the case of ipriflavone, which was recently demonstrated in vitro [8] and in vivo in an osteoporotic animal model [4]. 

In recent years, the role of osteoimmunology in osteoporotic processes has become evident [9]. Osteoimmunology explores the relationship between the skeletal and immune systems [10]. Cells in the immune system play a key role both in the development of disease and in their potential to stimulate therapeutic effects. Different effects of bone cells on immune regulation have been discovered, such as the role of osteoprogenitor cells in the regulation of hematopoietic stem cells and osteoblast-mediated suppression of hematopoietic neoplasms [11]. With regard to osteoporosis, osteoimmunology could be important in retarding or stopping osteolysis progression. Considering that the effect of estrogen insufficiency depends in part on T cells, it might be possible to change the immune activity in a favorable way [12].

The immune system regulates osteocytes by secreting inflammatory cytokines and other factors, which in turn affect bone formation and resorption. The immune and skeletal systems share the same microenvironment, and immune imbalance results in bone loss [13]. T and B lymphocytes and cytokines are important immune cells and regulatory factors, respectively, which affect bone resorption [14]. Specifically, various immune cells interact with osteoblasts and osteoclasts. Thus, specific subtypes of activated T lymphocytes express TNFα, IL-1, and IL-6, which indirectly stimulate osteoclastogenesis, triggering bone loss during osteoporosis. Th17 cells release interleukin-17 (IL-17), which induces the differentiation of mesenchymal stem cells into the osteogenic cell lineage, although it is true that it also indirectly increases osteoclast differentiation [15]. On the other hand, regulatory T lymphocytes (Treg cells) are suppressive agents of bone loss thanks to the inhibitory mechanism they exert on the differentiation of monocytes into osteoclasts. Therefore, it will be important to develop knowledge of osteoimmunology in order to identify new targets for immunotherapy of this pathology.

The potential impact of nanotechnology in the future treatment of osteoporosis using modulation of the osteoimmune response is being widely considered by different research groups [16,17]. However, most studies focus on the action that nanoparticles have on the polarization of macrophages toward M1 and M2 phenotypes. In the present work, we hypothesize that bioactive mesoporous nanoparticles loaded with ipriflavone can exert a positive osteoimmune effect, which goes beyond the polarization of macrophages toward reparative M2 phenotypes, as has been previously demonstrated [18]. However, the effect of these nanoparticles on the activation and behavior of T lymphocytes has not yet been addressed. CD4^+^ Th2 lymphocytes inhibit bone loss by significantly reducing the RANKL/OPG ratio through the secretion of cytokines [19]. Activated CD4^+^ Th2 lymphocytes are responsible for the formation and release of osteoprotegerin (OPG), which acts by blocking the interaction between receptor activator of nuclear factor κB receptor ligand (RANKL) and TNF-related apoptosis-inducing ligand (TRAIL)/APO2L and may exert a paracrine effect on adjacent cells, contributing to reducing the local process of bone remodeling and cell apoptosis [20]. Specifically, in the current study, we assessed in vitro studies on the effects of uncharged and ipriflavone-charged mesoporous bioactive glass nanoparticles, nanoMBGs and nanoMBG-IPs, respectively, on the CD4^+^ Th2 lymphocyte cell line SR.D10, which is considered as an ideal model for the study of the adaptive immune response. 

**Figure 1:**
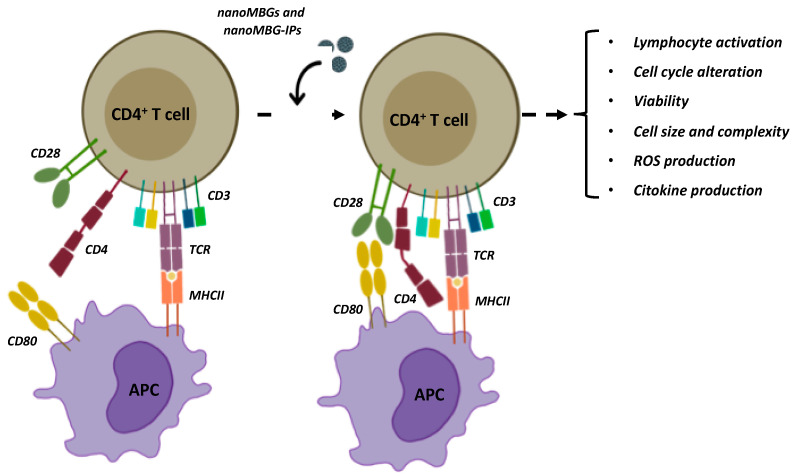
Interaction between an APC MCH II and a CD4^+^ T lymphocyte: TCR-MHC II interaction and the subsequent CD28-CD80 interaction and effects of uncharged and ipriflavone-charged mesoporous bioactive glass nanoparticles, nanoMBGs and nanoMBG-IPs, on different cellular parameters on CD4^+^ Th2 lymphocyte.

Our results show how the incorporation of these nanospheres by T helper lymphocytes does not alter their behavior (Figure 1), and this allowed us to better understand the functional state of Th cells after treatment with these potential nanocarriers. This study is aimed at demonstrating the action that this nanosystem has on the adaptive immune response through T lymphocytes and its ability to produce interleukins of anti-inflammatory nature. This fact could open new possibilities for the regenerative treatment of osteoporosis and fracture prevention.

## 2. Materials and Methods

### 2.1. Synthesis of Mesoporous Bioactive Nanoparticles

Bioactive mesoporous nanoparticles (nanoMBGs) were prepared following a synthesis methodology analogous to that previously published [4]. Briefly, nanoMBGs of nominal chemical composition 75SiO_2_-20CaO-5P_2_O_5_ (% mol) were prepared using the corresponding stoichiometric amounts of tetraethylortosilicate (TEOS), triethylphosphate (TEP), and calcium nitrate tetrahydrate (Ca(NO_3_)_2_·4H_2_O), using poly(styrene)-block-poly(acrylic acid) (PS-b-PAA) and hexadecyltrimethylammonium bromide (CTAB) as co-templates to introduce mesoporosity. PS-b-PAA was dissolved in tetrahydrofurane, whereas CTAB was dissolved in D.I. water and 2.4 mL of ammonia (28% *w*/*w*). Both solutions were mixed and, subsequently, TEOS, TEP, and calcium nitrate were added in one-hour intervals to the resulting microemulsion. After the hydrolysis and condensation process, the nanoparticles were collected using centrifugation and calcined at 550 °C for 4 h under air atmosphere. NanoMBGs were soaked in a highly concentrated IP solution in acetone for 24 h, subsequently washed with an aqueous solution of ethanol at 50%, and then filtered. A certain amount of nanoMBGs intended for assays where a fluorescent signal was required were labeled with fluorescein isothiocyanate (see Supporting Information for more detail). 

### 2.2. NanoMBGs Characterization 

Transmission electron microscopy (TEM) images were acquired with a JEOL-1400 microscope (Jeol Ltd., Tokyo, Japan). Fourier-transform infrared spectroscopy (FT-IR) was carried out with a Nicolet Magma IR 550 spectrometer, and thermogravimetric studies were performed using a Pyris Diamond TG/DTA thermobalance (Perkin Elmer, Waltham, MA, USA) between 30 °C and 600 °C under air atmosphere.

### 2.3. Culture of CD4^+^ Th2 SR.D10 Lymphocyte Cell Line

SR.D10 is a clonal variant produced in the laboratory of Dr. José María Rojo (Department of Cellular and Molecular Medicine, Centro de Investigaciones Biológicas Margarita Salas (CIB), CSIC, Madrid, Spain) and derived from the D10.G4.1 murine CD4^+^ Th2 cell line [21], that has specificity for the conalbumin/ovotransferrin fragment 134–146 linked to I–Ak class II major histocompatibility complex molecules of white chicken eggs. This cell line was cultured in Click’s medium, containing 10% heat-inactivated fetal calf serum (FCSi), 25 pg/mL mouse recombinant interleukin-1 (mrIL-1), 5 U/mL mrIL-2, and 10 U/mL mrIL-4 [22]. 

### 2.4. Treatment and Activation Assays of SR.D10 Lymphocytes with nanoMBGs and nanoMBG-IPs

CD4^+^ Th2 SR.D10 lymphocytes were grown at a concentration of 2.5 × 10^4^ cells/well in 96-well culture plates. They were cultured for 4 days at 37 °C under an atmosphere of 5% CO_2_ in Dulbecco’s Modified Eagle’s Medium (DMEM) containing 10% fetal bovine serum (FBS, Gibco, BRL), 200 μg/mL penicillin (BioWhittaker Europe, Verviers, Belgium), 200 μg/mL streptomycin (BioWhittaker Europe, Verviers, Belgium), and 1mM L-glutamine (BioWhittaker Europe, Verviers, Belgium) in the presence of interleukins. To carry out the activation assays, this cell line was kept for 2 h in the absence of ILs, and subsequently, they were maintained for 24 h in the absence or in the presence of 50 μg/mL of either nanoMBGs or nanoMBG-IPs, under both basal and stimulated conditions. Cell activation was achieved in vitro with the monoclonal antibodies Y-CD3-1 (anti-CD3ε) and GK1.5 (anti-CD4) (Sigma-Aldrich, St Louis, MO, USA), purified using affinity chromatography at 5 µg/mL, applied both individually and in combination to the SR.D10 lymphocytes for 24 h. After the treatment, media supernatants were compiled and kept at −20 °C until used for cytokine detection, and cell cultures were treated with Cell Counting Kit-8 (CCK-8, Sigma-Aldrich, St. Louis, MO, USA) between 2 and 4 h at 37 °C under 5% CO_2_ atmosphere in order to measure cell proliferation as an indirect indicator of lymphocyte activation [23]. A group of bare ipriflavone was not included in this study because of the lack of solubility of this drug in aqueous media. 

### 2.5. Effects of nanoMBGs on Cell Cycle Phases of SR.D10 Lymphocytes 

Cell suspensions were washed and incubated with 0.5 mL of PBS and 4.5 mL of ethanol 70% for 4 h at 4 °C. After 10 min and 310 g centrifugation, the cells were resuspended in 0.1% Triton X-100, 20 µg/mL propidium iodide (PI, Sigma-Aldrich, St. Louis, MO, USA), and 0.2 mg/mL of RNAsa (Sigma-Aldrich, St. Louis, MO, USA) and incubated at 37 °C for 30 min. PI fluorescence was detected by exciting the sample at 488 nm using a FACScalibur Becton Dickinson flow cytometer (Becton Dickinson, San Jose, CA, USA) with a 585/42 filter. The percentage of cells in each cell cycle phase was calculated using the CellQuest program (Becton Dickinson), using positive and negative controls and studying at least 10^4^ cells in each sample: SubG1, G0/G1, S, and G2/M (fractions indicative of apoptosis, growth, DNA synthesis, and growth/mitosis, respectively).

### 2.6. Cell Viability Studies of CD4^+^ Th2 SR.D10 Lymphocytes Evaluated using Flow Cytometry

Cell viability was analyzed by adding 20 µg/mL of propidium iodide (PI) into each cell suspension. Cells with damage in their plasma membrane incorporate the probe and the PI intercalates with their DNA. PI fluorescence was excited at 488 nm, and the emitted fluorescence was registered with a 530/30 filter with a FACScalibur Becton Dickinson flow cytometer (Becton Dickinson, San Jose, CA, USA).

### 2.7. Effects of nanoMBGs on the Size and Intracellular Complexity of SR.D10 Lymphocytes 

Cell size and complexity were evaluated after 15, 30, and 60 min of treatment with different concentrations of FITC-labeled nanoMBGs: 10, 30, and 50 µg/mL, using flow cytometry analyzing forward scatter (FSC) and side scatter (SSC), respectively, with a FACScalibur Becton Dickinson cytometer. Data acquisition and analysis conditions were performed with the CellQuest Program, analyzing a minimum of 10^4^ cells in each case.

### 2.8. Intracellular Content of Reactive Oxygen Species (ROS) in SR.D10 Lymphocytes 

After the treatment with nanoMBGs and nanoMBG-IPs for 24 h, the oxidative stress produced in SR.D10 lymphocytes was evaluated using an analysis of the intracellular content of reactive oxygen species (ROS). Each cell sample was incubated with 100 μM 2′,7′-dichlorofluorescein diacetate (DCFH/DA, Serva, Heidelberg, Germany) for 30 min at 37 °C. DCF fluorescence was excited at 488 nm and registered at 525 nm with a 530/30 filter using a FACScalibur Becton Dickinson. Data acquisition and analysis were performed with CellQuest, analyzing a minimum of 10^4^ cells in each case.

### 2.9. Mechanisms for the Incorporation of nanoMBGs by CD4^+^ Th2 SR.D10 Lymphocytes

Doses of 10, 30, and 50 μg/mL of FITC-labeled nanoMBGs were added to SR.D10 lymphocytes and maintained for different incubation times: 30, 60, and 90 min, to study the percentage of cells that incorporated nanoMBGs and the amount of nanomaterial they were able to incorporate. To identify the mechanisms by which the different cell types incorporated the nanoparticles, six specific inhibitors of different endocytic processes were added to the culture media, as shown in Table 1, and left to act for 90 min before the addition of nanoMBGs. 

After treatment with each inhibitor, 50 µg/mL of nanoMBGs were added for 30 min (without removing the inhibitor) and incubated in a 5% CO_2_ atmosphere at 37 °C. Cell suspensions were analyzed using flow cytometry with a FACScalibur Becton Dickinson cytometer. FITC fluorescence was excited at 488 nm and measured at 530/30 nm. A minimum of 10^4^ cells were analyzed in each case with CellQuest. 

### 2.10. Detection of Interleukins 4 (IL-4) and 10 (IL-10)

The measurement of cytokines (IL-4 and IL-10) secreted by the CD4^+^ Th2 SR.D10 lymphocytes was analyzed in the supernatants from the incubation of these cells under different conditions: without material and with 50 μg/mL nanoMBGs or nanoMBG-IPs. All cytokine detection assays consisted of an enzyme-linked immunosorbent assay (ELISA, Gen-Probe, Diaclone, Besançon, France). 

### 2.11. Data Analysis and Statistics

Results are shown as means ± standard deviations of three replications carried out at the same time in triplicate. Statistical analyses were performed using the Statistical Package for the Social Sciences (SPSS) version 22 program, using Student’s paired *t*-test comparisons to analyze the differences between pairs of groups and analysis of variance (ANOVA) to analyze the differences between more than two groups. Homogeneity of variances was assessed with Levene’s test, and differences between groups were assessed with the Scheffé or Games–Howell tests, depending on whether the variances were homogeneous or not, respectively. In all cases, statistical significance was considered for *p*-values < 0.05.

## 3. Results and Discussion

### 3.1. Characterization of nanoMBGs and nanoMBG-IPs

**Figure 2:**
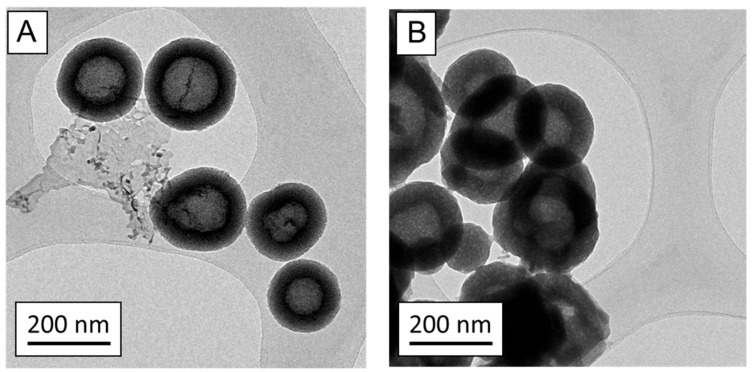
Transmission electron images of (**A**) nanoMBGs and (**B**) nanoMBG-IPs.

The TEM images of nanoMBGs (Figure 2A) show nanoparticles of 200 nm in diameter and exhibiting a hollow core–shell mesoporous structure, with an open radial porous structure in the shell component. After loading with IP (Figure 2B), the nanoMBG-IPs maintained this hollow core–shell structure, although the mesoporous structure appeared to be partially modified due to the drug loading process and filling of the mesopores with the drug. The amount of IP loaded into nanoMBGs was 17.25%, as determined using thermogravimetric analysis (see Supporting Information Figure S1). The presence of the drug was also confirmed using FTIR spectroscopy (see Supporting Information Figure S2) with the presence of new absorption bands corresponding to the chemical groups of IP and with the Si-O-Si stretching vibrational mode and the Si–O–Si bending mode at 1050 and 480 cm^−1^, respectively, that appear in both nanoMBG and nanoMBG-IP.

### 3.2. Uptake of nanoMBGs by Th2 CD4^+^ SR.D10 Lymphocytes

**Figure 3:**
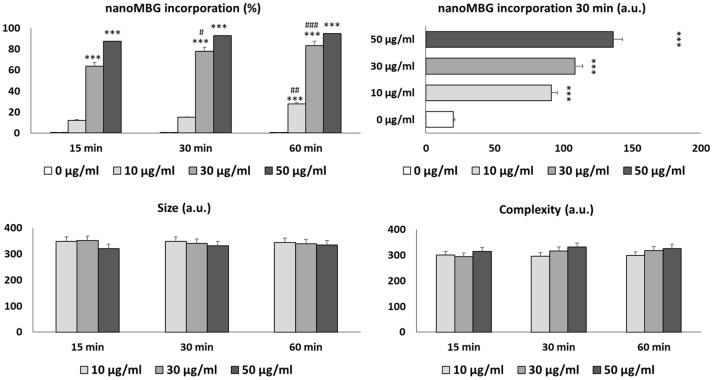
Incorporation of nanoMBGs, cell size and complexity of CD4^+^ Th2 SR.D10 lymphocytes. Statistical significance: *** *p* < 0.005 dose effect; # *p* < 0.05, ## p < 0.01, ### *p* < 0.005 time effect.

Figure 3 shows nanoMBGs incorporation by cultured CD4^+^ Th2 SR.D10 lymphocytes and its effects on cell size and complexity without inhibitors. The intracellular uptake of these nanospheres by T lymphocytes was time- and dose-dependent, in agreement with previous studies performed using different cell lines [18,24,25,26]. Thus, the percentage of cells that incorporated nanoMBGs after 15, 30, and 60 min was significantly superior (*p* < 0.005) in the presence of 30 and 50 μg/mL of nanospheres than after treatment with 10 μg/mL. The percentage of incorporation with 10 μg/mL of nanospheres during 15 and 30 min of exposure did not show statistically significant differences. However, a significant increment in this variable was observed with the same dose after 60 min.

Regarding the amount of incorporated nanoMBGs, SR.D10 lymphocytes did not saturate after 30 min of exposure, and, therefore, their exocytosis would not have taken place. As indicated by other authors, while endocytosis is essential for the targeting of nanoparticles in disease sites, exocytosis is the process of significance in the removal of nanoparticles from mammalian cells [27]. 

After the incorporation of nanoMBGs, no changes in lymphocyte size or complexity (FSC and SSC, respectively) were observed. These results are in concordance with those of previous studies on MC3T3-E1 preosteoblasts, in which the uptake of these nanospheres did not alter the above-mentioned parameters [24]. However, previous studies with another representative cell type of the innate immune system, RAW 264.7 macrophages, have shown a significant increment in cellular complexity (SSC) after treatment with these nanoparticles because of their incorporation by macrophages, probably due to the high phagocytosis capacity of this cell type [18].

### 3.3. Endocytic Strategies for the Uptake of nanoMBGs by Th2 CD4^+^ SR.D10 Lymphocytes

**Figure 4:**
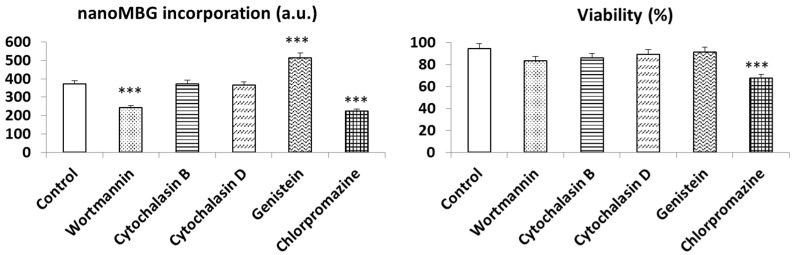
nanoMBG incorporation by Th2 CD4^+^ SR.D10 lymphocytes and their viability in the presence of endocytic inhibitors. Statistical significance: *** *p* < 0.005.

To understand the endocytic mechanisms involved in nanoMBGs uptake by cultured SR.D10 lymphocytes, five specific endocytosis inhibitors (wortmannin, cytochalasin B, cytochalasin D, genistein, and chlorpromazine) were added to the culture medium 30 min before nanoMBGs treatment. After contact with these specific inhibitors, cells were cultured for 90 min with 50 μg/mL nanoMBGs in the presence of each inhibitor, and the nanomaterial incorporation was analyzed using flow cytometry (Figure 4).

Previous studies using 50 µg/mL of these nanospheres with endothelial cells, preosteoblasts [24], and macrophages [18] made it possible to select this dose of nanomaterial as an optimal concentration without observing adverse effects. Thus, the results shown in Figure 3 evidence that the incorporation of these nanospheres by lymphocytes occurred in a dose-dependent manner, and the highest dose of 50 µg/mL did not alter the cellular parameters evaluated. Therefore, this concentration was selected and maintained for the analysis of endocytic mechanisms. These analyses were based on the fact that if the inhibitor of a specific endocytic process decreases the incorporation of the nanoparticle, it could indicate that this process was interrupted and could, therefore, be a route of entry. Previous experiments have made it possible to select the optimal dose of the various inhibitors used without compromising cell viability [28,29].

The treatment with wortmannin, a specific inhibitor of phagocytosis via phosphoinositide 3-kinase, and chlorpromazine, an inhibitor of clathrin-mediated endocytosis mechanisms, decreased the incorporation of nanoMBGs by SR.D10 lymphocytes. This fact indicates that uptake is mediated by mechanisms that involve PI3-kinase and clathrin-mediated processes. However, cytochalasin B or D, which are inhibitors of macropinocytosis, had no effect on the uptake of nanoMBGs by lymphocytes under the doses and test conditions used. Conversely, there was a significant increase in nanoMBGs entry in the presence of genistein, suggesting that caveolae-mediated endocytosis is not implicated in the mechanism for nanoMBGs uptake by this cell type. Genistein inhibits multiple tyrosine kinases of the Src family, which affects various processes such as endocytosis or chemotactic signaling, and, therefore, the dynamics of caveolae [30]. Recently, the importance of caveolin 3 expression in lymphocyte activation and differentiation was demonstrated [31]. In addition, genistein is directly involved in actin polymerization in CD4^+^ Th2 memory cells [32], and this alteration could favor the uptake of nanoMBGs by SR.D10 lymphocytes. The same effect was observed using Saos-2 bone cells in the presence of graphene oxide nanoparticles after treatment with the above-mentioned antibiotic [29]. However, this effect was not observed in myeloid dendritic DC2.4 cells, so it can be concluded that the differences in the internalization mechanisms of nanoMBGs in different cell lines could be due to the different origin of the cells, myeloid or lymphoid, to the different expression of surface receptors, or to the distinct sensitivity to the specific inhibitors [26]. 

Certain compounds with flavonoid skeletons, such as the drug ipriflavone encapsulated in nanoMBG, have endocytosis-inhibitory properties by themselves. However, previous studies have demonstrated that the effects of these nanospheres loaded with ipriflavone on different cellular parameters such as viability, reactive oxygen species production, and the cell cycle, are similar to nanoMBGs effects, and they also enhance osteogenesis, measured with MC3T3-E1 pre-osteoblasts differentiation, as well as endothelial progenitor cell differentiation towards mature phenotypes [8,12,24]. All these previous studies demonstrate their incorporation by cells, drug release, and effective drug action.

Figure 4 also shows the effects of these specific endocytic inhibitors and nanoMBGs on the cell viability of CD4^+^ Th2 lymphocytes, comparing them with controls without inhibitors and without nanomaterials, performed in parallel. Although high viability values were obtained with this lymphocyte line after these treatments, chlorpromazine induced a significant decrease in this parameter, which confirms certain cytotoxicity of this compound [24].

### 3.4. Impact of Nanoparticles (MBGs and MBG-IPs) on Cell-Cycle Phases of CD4^+^ Th2 Memory Cells

Naive lymphocytes are quiescent and need adequate activation to progress through the cell cycle. In response to antigenic stimulation, lymphocytes will proliferate, differentiate, and achieve their effector functions, and most activated T cells will die by a mechanism of apoptosis. Thus, the cell cycle has an important role in the differentiation of T cells and cell death induced by incorrect activation [33,34].

**Figure 5:**
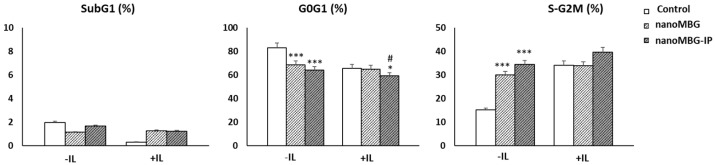
Effect of nanoMBGs and nanoMBG-IPs on the cell cycle phases of CD4^+^ Th2 SR.D10 lymphocytes: SubG_1_ (indicative of apoptosis), G_0_/G_1_ (quiescence/gap1), and S/G_2_/M (synthesis/gap 2/mitosis). +IL = interleukin-enriched medium (25 pg/mL mrIL-1, 5 U/mL mrIL-2, and 10 U/mL mrIL-4), −IL = medium without interleukins. Statistical significance: * *p* < 0.05, *** *p* < 0.005 conditions vs. control; # *p* < 0.05 nanoMBGs vs. nanoMBG-IPs).

Using flow cytometry analysis, we studied the effect on cell-cycle phases (G_0_/G_1_, S-G_2_/M) of CD4^+^ T helper lymphocytes in the presence of unloaded and ipriflavone-loaded nanospheres (nanoMBGs and nanoMBG-Ips, respectively) both in the absence and presence of interleukins. SubG_1_ fraction, which corresponds to cells with fragmented DNA, was evaluated as an indicator for apoptosis. Figure 5 shows that treatment with 50 μg/mL of nanospheres without ipriflavone for 24 h in the absence of interleukins induced a decrease in the percentage of cells in the G_0_/G_1_ phase, accompanied by an increase in the S/G_2_/M phase (*p* < 0.005). This effect was more pronounced with the IP-loaded nanospheres. This is consistent with results achieved in previous studies with MC3T3-E1 preosteoblasts, in which the incorporation of these nanospheres induced a significant increment in the synthesis phase (S) [24]. However, in the presence of a culture medium rich in interleukins, no alterations were observed in any of the phases, just a slight decrease in cells in the G_0_/G_1_ phase (*p* < 0.05).

These results show the relevance of this interleukin-enriched culture medium in the proliferation of these cells, in agreement with previous results for the group with murine CD4^+^ Th2 SR.D10 lymphocytes in the presence of graphene oxide nanostructures [35]. Moreover, nanoMBGs and nanoMBG–IPs did not trigger lymphocyte apoptosis, detected as SubG_1_ fraction, in comparison with control cultures, neither in the absence nor presence of interleukins.

It should be noted that no significant differences were detected between unloaded (nanoMBGs) and ipriflavone-loaded nanospheres (nanoMBG-IPs) in the resting cycle phase (G_0_/G_1_) and in the proliferation phase (S/G_2_/M), suggesting that the intracellular release of ipriflavone does not activate lymphocytes since these are not its target cell. The target cells of this drug are osteoblasts, osteoclasts, and osteoprogenitor cells [18,24]. This result supports the absence of apoptosis observed, and it would contribute to decreasing the inflammatory process in the case of osteoporosis. 

### 3.5. Proliferation of CD4^+^ Th2 SR.D10 Lymphocytes after Treatment with nanoMBGs and nanoMBG-IPs in Basal and Stimulated Conditions

**Figure 6:**
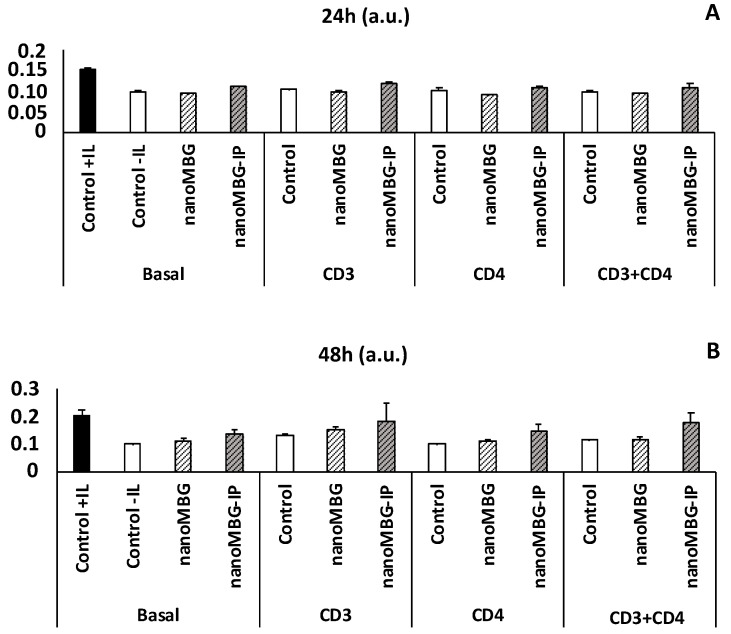
Effect of nanoMBGs and nanoMBG-IPs on the proliferation of Th2 CD4^+^ SR.D10 lymphocytes in basal and activated conditions with CD3, CD4, or both, after 24 h (**A**) and 48 h (**B**). No statistically significant differences were observed.

SR.D10 lymphocytes were stimulated for 24 and 48 h (Figure 6A and 6B, respectively) with YCD3-1 (anti-CD3) and GK1.5 (anti-CD4) or anti-CD3+CD4 monoclonal antibodies in the absence and presence of nanoMBGs and nanoMBG-IPs. As shown in Figure 6, lymphocytes responded adequately to all stimuli in the absence and presence of these nanospheres; therefore, their presence did not significantly modify the activation conditions of lymphocytes. We only observed a slight significant increase in the case of nanoMBG-IPs in basal conditions at both exposure times, as well as after stimulation with the CD4 co-receptor at 48 h.

As predicted, in basal conditions without stimuli, in the absence of an interleukin-rich medium (-IL), the lymphocyte proliferation was lower than in the presence of an interleukin-rich medium (+IL) [35].

T-lymphocyte proliferation is a necessary process for an adequate immune response to occur. Furthermore, the activation of lymphocytes causes differentiation mechanisms that will determine the type of cell they will develop into and the particular cytokines they will release [36,37]; therefore, T-lymphocyte activation and proliferation are two interrelated processes. The first signaling is mediated by an interaction with the T cell receptor (TCR-CD3), which recognizes small antigenic peptides, associated with major histocompatibility complex (MHC) molecules on the macrophage plasma membrane and other antigen-presenting cells (APCs), such as ligands. CD4 or CD8 co-receptors assist this process on the T cell, which stabilizes TCR-mediated interactions and enhances intracellular communication (Figure 1). The second signal is mediated by the B7 family (CD80, CD86) co-stimulatory molecules present on APCs, which act as ligands for CD28, the main co-stimulatory molecule on the T cell. This specific recognition leads to T cell proliferation with the capacity to recognize the antigen. The third signal corresponds to the signaling mediated by APC-secrete effector cytokines, which are determinants in the differentiation of effector T cells.

In the current study, the effects of nanoMBGs and nanoMBG-IPs on Th2 lymphocyte proliferation were examined after the activation of the TCR-CD3 receptor (T cell antigen receptor) and the CD4 surface receptor (main T helper cell co-receptor) and by co-stimulation with TCR/CD3 and CD4 (Figure 1). The name SRD10 cells “syngeneic-reactive D10” is associated with their origin as they were isolated from a mouse CD4+ helper T lymphocyte clone, D10 G4.1 (D10). This cell line is therefore a differentiated line of CD4+ Th2 lymphocytes with the capacity to respond to both TCR-dependent and TCR-independent stimuli (hyperreactivity), indicating that it has a lower activation response threshold than the original clone.

This fact, together with the possibility of growing them in a medium supplemented with interleukins, makes this cell line a very useful experimental model for analyzing T cell responses [36]. For this reason, to perform these activation assays, cells cultured in the interleukin-rich medium were exhaustively washed prior to stimulation with the above-mentioned antibodies.

It is important to highlight that there were no significant differences in lymphocyte activation in any of the conditions used in this study, either in the presence of unloaded (nanoMBGs) or ipriflavone-loaded nanospheres (nanoMBG-IPs). Therefore, the use of ipriflavone as an anti-osteoporotic drug could be very useful for the treatment of osteoporosis without producing alterations in the lymphocyte response, which is why research in this direction could allow us to find more therapeutic molecular targets to address this disease.

### 3.6. Effects of nanoMBGs and nanoMBG-IPs on Cell Viability and Intracellular Reactive Oxygen Species (ROS) Content in CD4^+^ Th2 SR.D10 Lymphocytes in Basal and Stimulated Conditions

While no negative effects on the cell cycle were observed and lymphocytes responded adequately to all activating stimuli, both in the absence and the presence of nanoMBGs and nanoMBG-IPs, the incorporation of nanoparticles could trigger an increase in the intracellular content of reactive oxygen species (ROS) and the induction of oxidative stress, which are the main toxicity mechanisms proposed for nanomaterials [38]. Numerous papers have been published on oxidative stress induced by various types of nanomaterials and their importance in relation to components of the immune system [39]. On the other hand, these bioactive mesoporous nanospheres are made of silicon, calcium, and phosphorous oxides, whose ions play an important role in cell proliferation, homeostasis, and bone remodeling [40]. 

**Figure 7:**
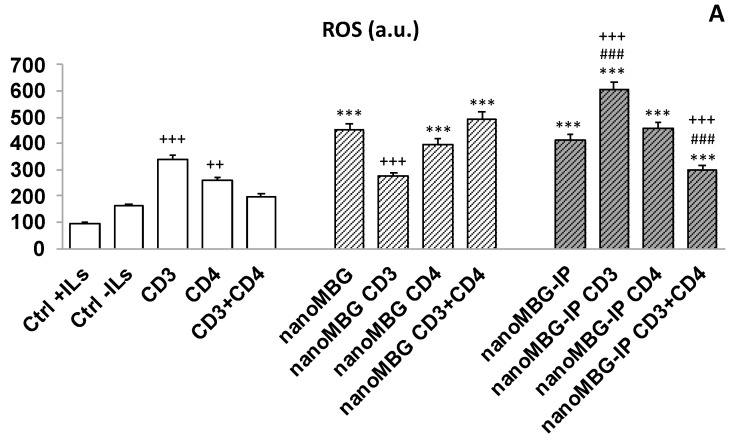

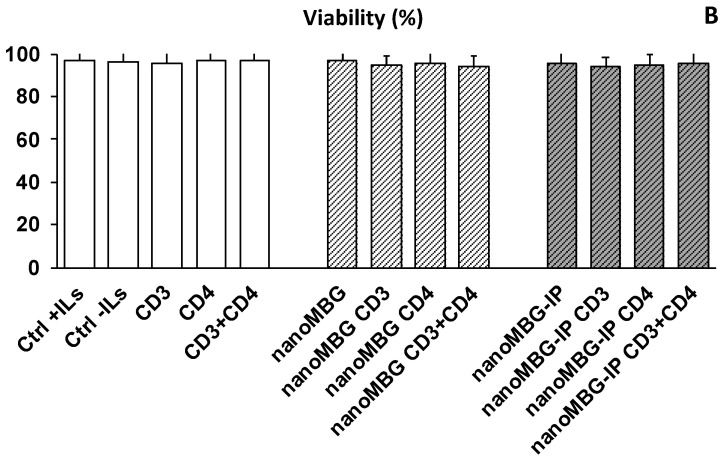
Effect of nanoMBGs and nanoMBG-IPs on the intracellular content of reactive oxygen species (ROS) (**A**) and viability (**B**) of Th2 CD4^+^ SR.D10 lymphocytes in basal and activated conditions with CD3, CD4, or both. Statistical significance: *** *p* < 0.005 Ct vs. Sph+F or Sph-F in each condition; ### *p* < 0.005 Sph-F vs. Sph+F in each condition; ++ *p* < 0.01, +++ *p* < 0.005 stimuli effect.

Considering all these facts, we evaluated the cell viability and intracellular reactive oxygen species (ROS) content in CD4+ Th2 SR.D10 lymphocytes after 24 h of treatment with nanoMBGs and nanoMBG-IPs. These experiments were carried out both under basal conditions and specific activation of the T cell receptor (TCR-CD3), the CD4 surface receptor, the main co-receptor of CD4+ Th2 lymphocytes, and simultaneous stimulation with the two receptors, TCR-CD3 and CD4. The control cells were cultured in the presence of stimuli without material (SR.D10 CONTROL) in parallel following the same experimental method. The results are shown in Figure 7.

In the absence of the nanospheres, the intracellular ROS content increased significantly after stimulation with the TCR/CD3 and CD4 lymphocyte receptor when monoclonal antibodies YCD3-1 (anti-CD3ε) [23] and GK1.5 (anti-CD4) [41] were added to the T lymphocytes. When the lymphocyte line received the two stimuli at the same time (CD3 + CD4), the observed effect was the opposite, a fact that was also observed previously for murine SR.D10 lymphocytes in the presence of graphene oxide nanostructures. [35].

On the other hand, treatment with 50 μg/mL of nanospheres without ipriflavone (nanoMBGs) for 24 h cultured in the absence of ILs induced an increase in the intracellular ROS that was modulated in the presence of stimuli through the main receptors of T lymphocytes, which was significantly appreciable in the case of stimulation with TCR/CD3. In the case of treatment in the presence of nanospheres with ipriflavone (nanoMBG-IPs), a similar pattern was observed, that is, a significant increase in levels of ROS after the activation under stimulation with CD3 and a reversal of the effect when the line of lymphocytes received stimulation with TCR/CD3 together with CD4 (Figure 7A). 

It has been widely demonstrated that proper activation of a virgin T lymphocyte requires another interaction between its receptor (TCR/CD3) and co-receptor (CD4 and CD8) molecules and the ligands presented in the professional mature APCs (carriers of the antigenic peptides) [41,42,43]. During the maturation process, one of these surface glycoproteins is selected, resulting in TCR/CD4^+^ or TCR/CD8+ cells. CD4+ cells specifically express a TCR that recognizes antigens presented by the major histocompatibility complex class II (MHC II) and exerts mainly helper functions. These interactions contribute to the formation of the trimolecular MHC/TCR/CD4 complex (Figure 1), stabilizing TCR-mediated interactions and promoting intracellular communication by activation of p56lck kinase, a CD4 intracellular amino-terminal region-associated kinase. This signal, together with the activation of protein tyrosine kinases (PTKs) associated with the TCR-CD3 complex, such as p59fyn, ZAP-70, and p72syc kinases, causes an efficient signaling cascade necessary for the correct activation of the T lymphocyte [43,44].

The anti-TCR/CD3 antibodies used in this study induced an association between CD4 and the TCR complex on CD4^+^ T cells [37], and this association is necessary for correct lymphocyte activation. This aspect is important as it ensures that T cells will only be activated when they recognize antigenic peptides presented in a class II MHC context by antigen-presenting cells (APCs), which have previously recognized and processed pathogens, together with CD4 co-receptor stimulation, thus preventing the lymphocyte from an inappropriate response.

Reactive oxygen species (ROS) act as an intracellular regulator when generated in a controlled manner at specific points in the cell, thus modifying protein function through the reversible oxidation of cysteines, regulating protein kinases and phosphatases, transcription factors, and ion channels [45]. In addition, ROS have been shown to interfere with signaling cascade systems related to cell proliferation, including the activation of nuclear transcription factor NF-ĸB, activator protein 1 (AP-1), phospholipase-A2, MAPKs, and c-Jun kinase, which are crucial for the expression of effector molecules required for lymphocyte function [46]. On the other hand, there is a relationship between the production of reactive oxygen species and increased resorptive activity of osteoclasts [47] while generating apoptosis in the osteoblasts [48]. Both effects lead to a loss of bone mass. In this study, we observed that lymphocytes increased their ROS production after treatment with nanoMBGs, but when ipriflavone was loaded inside the nanospheres, the ROS content decreased (Figure 7A). When CD3 and CD4 simultaneously stimulated the lymphocyte, this effect was enhanced, which would imply that ipriflavone reduces the inflammatory state of the lymphocyte and thus the activation of the osteoclast, which is essential in an osteoporotic scenario.

These results agree with the antioxidant and anti-inflammatory effects of ipriflavone observed by other authors [49]. Furthermore, nanoMBGs and nanoMBG-IPs did not induce changes in the viability of lymphocytes, detected as a percentage of live cells, compared to control cultures, either in the absence or presence of interleukin-rich medium or in any of the activation situations (Figure 7B). All these results show that, despite the increase in ROS in some of the conditions tested, the uptake of nanoMBGs and nanoMBG-IPs does not significantly modify the activation conditions of lymphocytes or affect cell viability.

### 3.7. Effects of nanoMBGs and nanoMBG-IPs on Interleukin-4 (IL-4) and Interleukin-10 (IL-10) Secretion by CD4^+^ Th2 SR.D10 Lymphocytes in Basal and Stimulated Conditions

**Figure 8:**
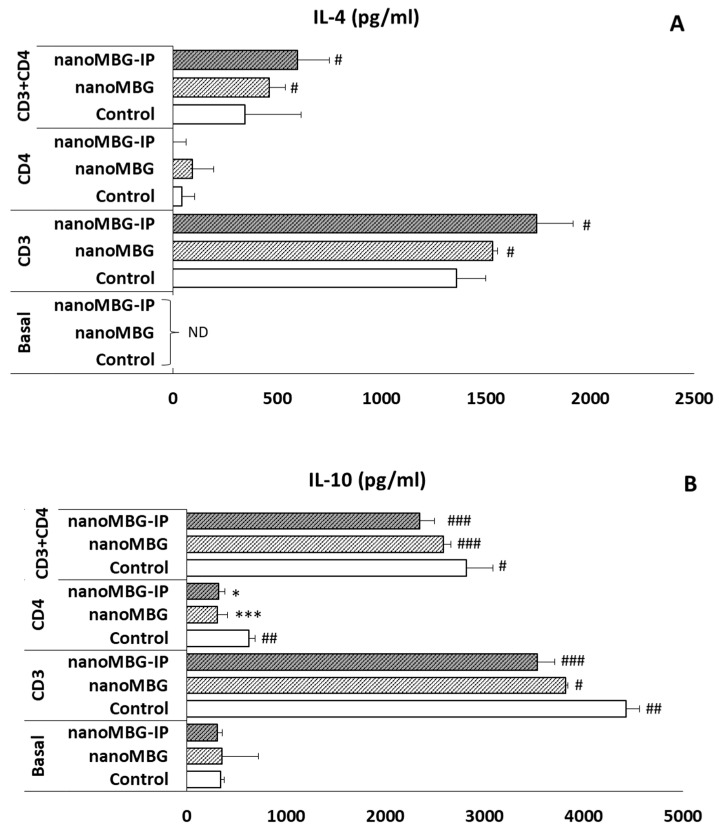
Effects of nanoMBG and nanoMBG-IP on the production of IL-4 (**A**) and IL-10 (**B**) by Th2 CD4^+^ SR.D10 lymphocytes under basal and stimulated conditions with CD3, CD4, or both. ND = Non-detected. Statistical significance: * *p* < 0.05, *** *p* < 0.005 conditions vs. control; # *p* < 0.05, ## *p* < 0.01, ### *p* < 0.005 stimulus effect.

In the present study, the levels of IL-4 and IL-10, which are anti-inflammatory intracellular interleukins, were quantified to thoroughly study the potential effect of nanoMBGs and nanoMBG-IPs nanospheres on anti-inflammatory cytokines secretion. SR.D10 lymphocytes were cultivated with nanoMBGs and nanoMBG-IPs under basal and stimulated conditions. As can be observed in Figure 8, under basal conditions and after 24 h of incubation with these nanospheres, there were no significant effects of nanoMBGs or nanoMBG-IPs on the secreted levels of IL-4 (Figure 8A) or the anti-inflammatory IL-10 (Figure 8B). Upon analysis of the IL-4 levels in the supernatants of stimulated lymphocytes, SR.D10 activated either with the TCR/CD3 receptor or TCR/CD3+CD4 showed a significant increase both in the presence of nanoMBGs and nanoMBG-IPs, compared to control cells without material cultured in the absence of ILs (Figure 8A). This increase was more pronounced when stimulated in the presence of nanoMBG-IPs, in agreement with the anti-inflammatory effect of ipriflavone observed by other authors [49,50]. These results indicate that the secretion of this anti-inflammatory regulatory cytokine IL-4 increases after the uptake of nanoMBGs and nanoMBG-IPs, favoring the secretion of this cytokine in the case of adequate stimulation of the SR.D10 lymphocytes with their receptor and co-receptor. 

On the other hand, as can be seen in Figure 8B, the levels of IL-10 in the supernatants were significantly modified, compared to the controls, in the absence of nanomaterial. Significant increases in IL-10 were detected in the control cells at all stimulation conditions (## *p* < 0.001, * *p* < 0.05). However, after the incorporation of nanoMBG and nanoMBG-IP nanospheres, a significant decrease was observed.

Although nanospheres do not produce cytotoxicity or affect lymphocyte activation, their potential inflammatory effect should be assessed. In this context, several cells involved in innate immunity are important regulators of immunoporosis. Cells in the innate immune system modulate osteoporosis by producing various proinflammatory mediators and largely by affecting the RANK/RANKL/OPG pathway. Osteoclasts, which are responsible for bone resorption, share a common origin with some major innate immune cells, allowing them to have several features that overlap, such as the expression of a common set of PRRs, the production of several proinflammatory cytokines and their receptors, providing an effective nexus of information between the skeletal and immune systems [51].

Furthermore, it was recently demonstrated that mouse peritoneal macrophages polarize into an M2 type producing regulatory cytokines, such as IL-4, IL-13, TGF-β, and IL-10, and could suppress T-cell proliferation in vitro. Polarization to M1/M2 and macrophage functions are tightly regulated through the activation of distinct interconnected pathways. It has been shown that the balance between STAT1 and STAT3/STAT6 activation plays a crucial role; therefore, prevalent STAT1 activation induces the polarization of M1 macrophages, which exert cytotoxic and proinflammatory functions. In contrast, STAT3 and STAT6 activation mediated by IL-4/IL-13 and IL-10 signaling stimulates macrophage polarization towards the M2 phenotype, which is associated with active immunotolerance and tissue repair [46,52,53,54]. IL-10 is a strong anti-inflammatory cytokine that suppresses both immunoproliferative and inflammatory responses, playing an important role in bone and joint diseases, including osteoporosis. Therefore, it is essential to understand the relationship of IL-10 in bone loss-related diseases [55]. T helper 2 cells produce IL-10, which may play an inhibitory role in the production of cytokines by T helper 1 cells [56]. In the case of IL-4, it plays an important role in the regulation of bone homeostasis since it suppresses RANKL-induced osteoclast differentiation by acting directly on osteoclast precursors. [57,58]. In this context, a reduction in IL-4 was observed when a dietary supplement such as *Dioscorea alata* (antioxidant and anti-diabetic with anti-osteoporotic properties) was incorporated. The antioxidant agent acts at the level of the immune system by polarizing the Th0 lymphocyte population towards the expression of the Th1 immune response by up-regulating the expression of IFN-γ and IL-2 and down-regulating the expression of IL-4 and IL-10 [19].

Th2 lymphocytes inhibit bone loss by reducing the RANKL/OPG ratio through cytokine secretion [59]. In this regard, IL-4 inhibits bone resorption, with low levels of Th2 cytokines IL-4 and IL-10 being observed in the synovial fluid and peripheral blood of patients with osteoarthritis [60]. Thus, these facts, together with the fact that IL-4 promotes Th2 polarization of native CD4+ T cells in vitro [61], confirm the activity of Th2 lymphocytes as bone protective agents, which is of particular relevance in osteoporosis scenarios.

Finally, particle size and morphology play a fundamental role in the inflammatory response they induce. In the more specific field of SiO_2_-based nanoparticles (as is the case in this research), it has been shown that particles with sizes below 1000 nm induce an inflammatory response superior to that produced by particles of larger sizes, causing pulmonary inflammation when administered intratracheally [62]. However, particle morphology and composition also determine the magnitude of the inflammatory response. Thus, Lebre et al. demonstrated that needle-shaped particles of 0.1 μm induce a strong inflammatory response after intraperitoneal injection, which is not observed with spherical particles of similar size [63]. Indeed, our results indicate that mesoporous SiO_2_ -CaO nanoparticles do not affect T cell viability or function, nor do they induce inflammatory mediators or oxidative stress. The small size of nanoMBGs (around 200 nm) could induce this type of response. However, the spherical shape and the greater biodegradability due to the presence of Ca^2+^ cations would contribute to their greater biocompatibility compared to acicular nanoparticles or to those composed exclusively of SiO_2_, as has been previously proven with in vivo studies in osteoporotic rabbit models [4].

## 4. Conclusions

The results obtained in this study demonstrate that the incorporation of mesoporous bioactive nanoparticles does not affect the viability or function of T helper lymphocytes, nor induce inflammatory mediators or oxidative stress, and maintains the reparative phenotype characteristic of Th2 lymphocytes involved in tissue repair, indicating that the immune response to these nanoparticles is adequate. Moreover, the incorporation of the antiosteoporotic drug ipriflavone contributes to the decrease in a potential unwanted inflammatory response by decreasing the presence of ROS and stimulating the secretion of IL-4. In conclusion, this work contributes to a better understanding of the functional status of T helper lymphocytes after treatment with these nanocarriers, proving that these bioactive mesoporous glass nanospheres loaded with ipriflavone can exert a positive osteoimmune effect.

## Figures and Tables

**Table 1:** Effects of endocytosis inhibitors including the dose used, molecular mechanisms affected, and molecular target.

Inhibitor	Concentration	Mechanisms	Target
Cytochalasin B	20 µM	Macropinocytosis	Blockage of actin polymerization
Cytochalasin D	4 µM	Macropinocytosis	Blockage of actin polymerization and other endocytosis routes
Chlorpromazine	30 µM	Clathrin-mediated endocytosis	Alteration in the fluidity and permeability of the membrane and the assembly of clathrin-coat
Genistein	3.7 µM	Non clathrin-mediated endocytosis	Blockage of caveolae dynamics and inhibition of Src tyrosine kinases
Phenylarsine oxide (PAO)	3.7 µM	Clathrin-mediated endocytosis	Blockage of tyrosine phosphatases decreasing membrane fluidity
Wortmannin	23 µM	Phagocytosis	Blockage of kinases (as PI3K)

## Data Availability

The data presented in this study are available on request from the corresponding author M.J.F.

## Supplementary Materials

The following supporting information can be downloaded at: https://www.mdpi.com/article/10.3390/nano13152183/s1, Labeling of nanoMBGs with fluorescein isothiocyanate; Figure S1: Thermogravimetric analysis of nanoMBGs and nanoMBG-IPs; Figure S2: FTIR spectra collected from nanoMBGs and nanoMBG-IPs and ipriflavone (IP).
