# Supplementary material for: Osteoimmune Properties of Mesoporous Bioactive Nanospheres: A Study on T Helper Lymphocytes
Source: Nanomaterials (Basel). 2023 Jul 26;13(15):2183. doi: 10.3390/nano13152183 (PMC10421130; doi:10.3390/nano13152183)
Supplement: Supplementary file 1 [file nanomaterials-13-02183-s001.zip › nanomaterials-2500759-supplementary.pdf]

Osteoimmune Properties of Mesoporous Bioactive Nanospheres: A Study on T Helper Lymphocytes

Laura Casarrubios [1,2], Mónica Cicuéndez [2,3], María Vallet-Regí [3,4], María Teresa Portolés [1,2,5], Daniel Arcos [3,4,5,*] and María José Feito [1,2,*]

1 Departamento de Bioquímica y Biología Molecular, Facultad de Ciencias Químicas, Universidad Complutense de Madrid, 28040 Madrid, Spain
2 Instituto de Investigación Sanitaria del Hospital Clínico San Carlos (IdISSC), 28040 Madrid, Spain
3 Departamento de Química en Ciencias Farmacéuticas, Facultad de Farmacia, Universidad Complutense de Madrid, 28040 Madrid, Spain
4 Instituto de Investigación Sanitaria Hospital 12 de Octubre i+12, 28040 Madrid, Spain
5 CIBER de Bioingeniería, Biomateriales y Nanomedicina, CIBER-BBN, ISCIII, 28040 Madrid, Spain

Supporting information

Labeling of nanoMBGs with fluorescein isothiocyanate

Aminopropyl triethoxysilane (APTES) was dissolved in ethanol. Subsequently, 0.6 mg of fluorescein isothiocyanate were added and stirred for 5 h. This solution was added dropwise into the nanoMBG particles suspension, and the labeled particles were washed and collected by centrifugation.

Ipriflavone loading determination

Thermogravimetrical analysis was carried out on both nanoMBG and nanoMBG-IP samples. In both cases there is a weight loss between 30 °C and 100 °C corresponding to the water absorbed on the porous surface. In addition, the condensation of silanol (Si-OH) groups also lead to an additional water desorption that was calculated to be around 3.25% in both cases. The thermogram of nanoMBG-IPs shows a loss of weight of 17.25 % between 250 °C and 500 °C that corresponds to the amount of IP loaded within nanoMBG-IPs.

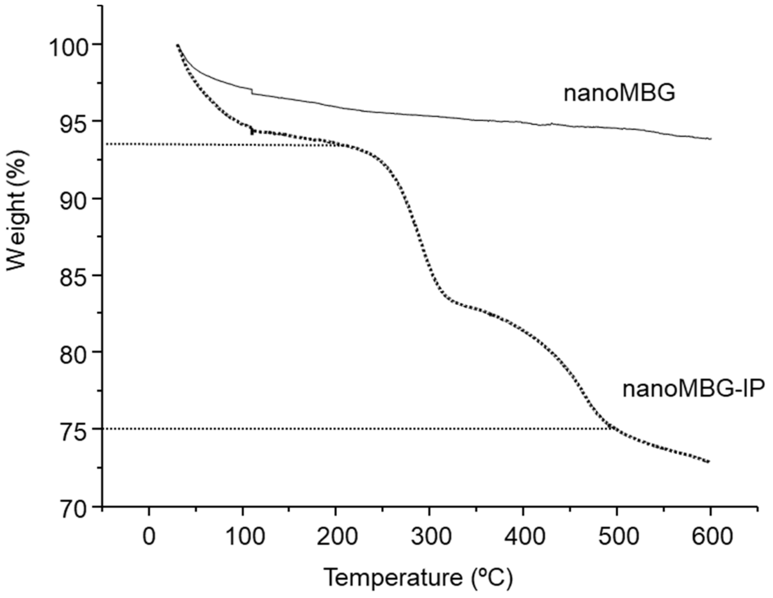

Figure S1. Thermogravimetrical analysis of nanoMBGs, nanoMBG-IPs

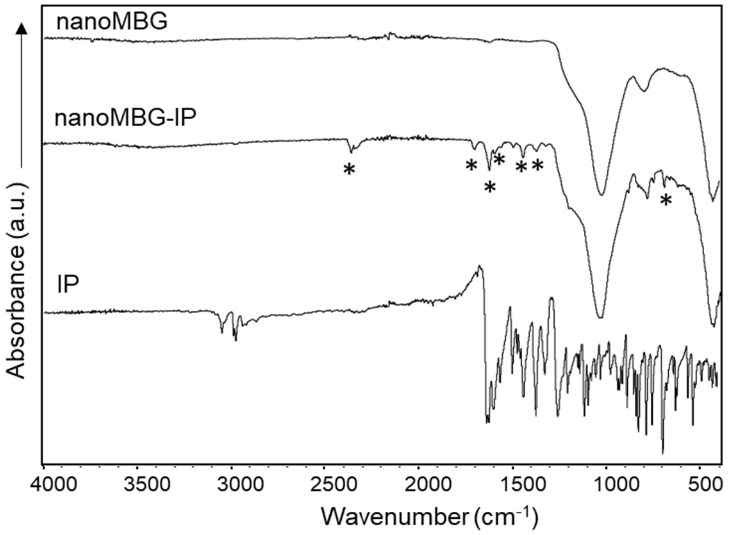

Figure S2. FTIR spectra collected from nanoMBGs and nanoMBG-IPs and ipriflavone (IP). (*) indicates absorption bands corresponding to ipriflavone functional groups.
